# Supplementary material for: Dynamic X-ray Microtomography vs. Laser-Doppler Vibrometry: A Comparative Study
Source: J Assoc Res Otolaryngol. 2025 Jan 14;26(1):63–75. doi: 10.1007/s10162-024-00971-0 (PMC11861830; doi:10.1007/s10162-024-00971-0)
Supplement: Supplementary file 1 — Supplementary file1 (DOCX 1023 KB) [file 10162_2024_971_MOESM1_ESM.docx]

**Supplementary Information**


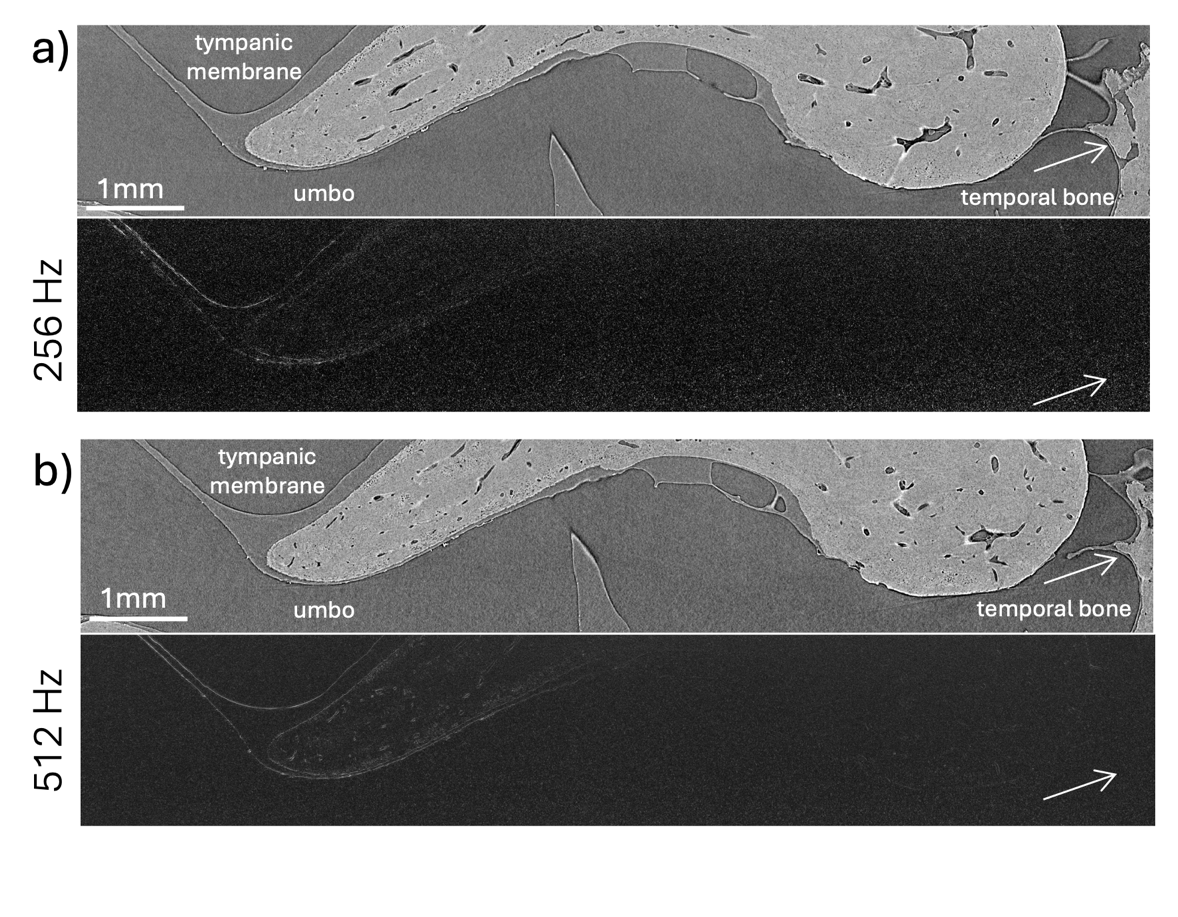


**Supplementary Fig. 1 2D visualizations of the malleus in TB1 stimulated at 120 dB SPL for 256 Hz and 512 Hz.** The top images in (a) and (b) display vertical reslices of the malleus (TB1), stimulated at 256 Hz (a) and 512 Hz (b), respectively, at 120 dB SPL. These reslices are taken from each reconstructed 3D set of volume across all 10 phases of movement. The ten resliced images of the same region are then projected into 2D using a standard deviation projection, which highlights the moving features shown in the bottom images of (a) and (b). The white arrows indicate parts of the temporal bone that remain static during sound stimulation.
