# Supplementary material for: Dynamic X-ray Microtomography vs. Laser-Doppler Vibrometry: A Comparative Study
Source: J Assoc Res Otolaryngol. 2025 Jan 14;26(1):63–75. doi: 10.1007/s10162-024-00971-0 (PMC11861830; doi:10.1007/s10162-024-00971-0)

Supplementary Material

Motion Quantification Graphs for all TBs

Corresponding author: aleksandra.ivanovic@unibe.ch

TB1

256 Hz – 110 dB SPL

stapes

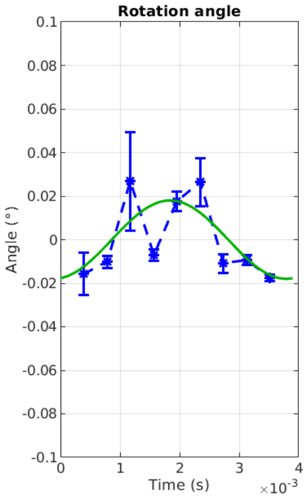

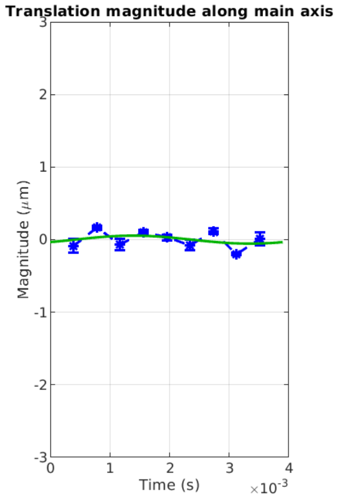

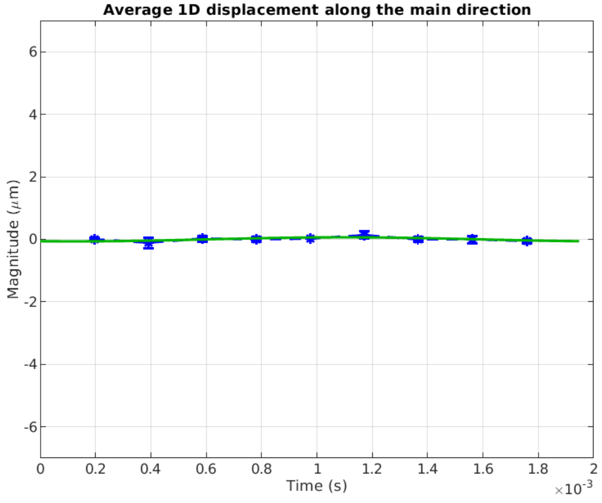

malleus

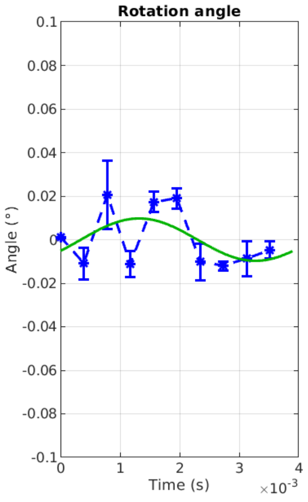

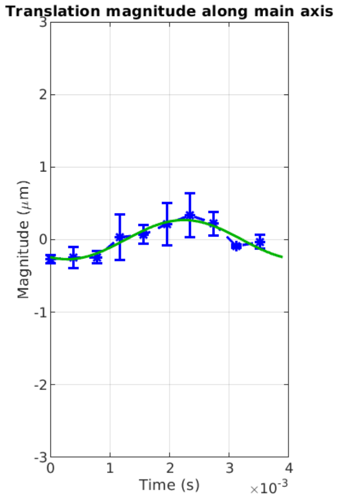

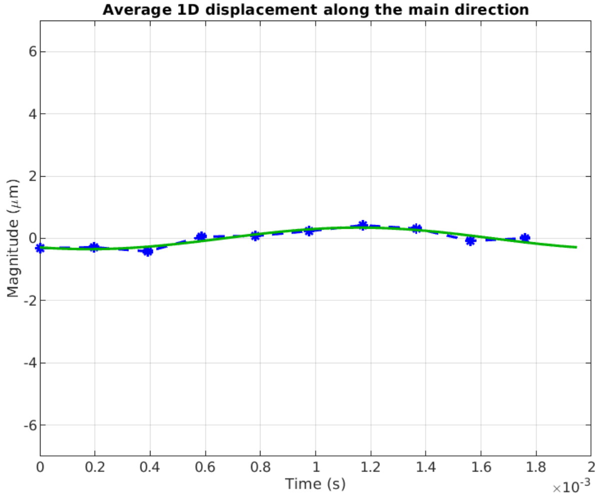

TB1

256 Hz – 120 dB SPL

stapes

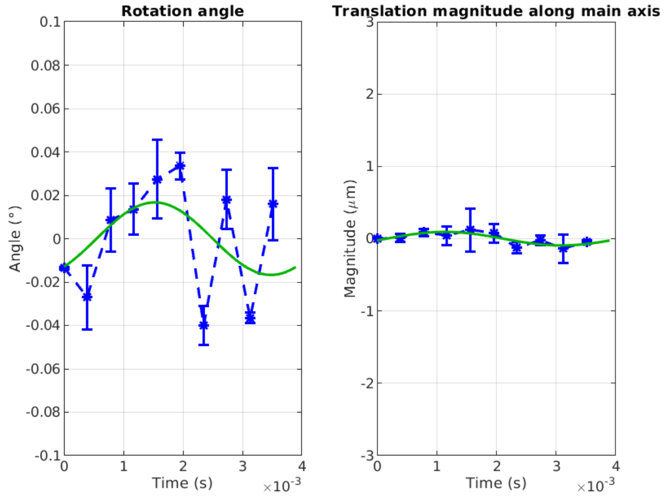

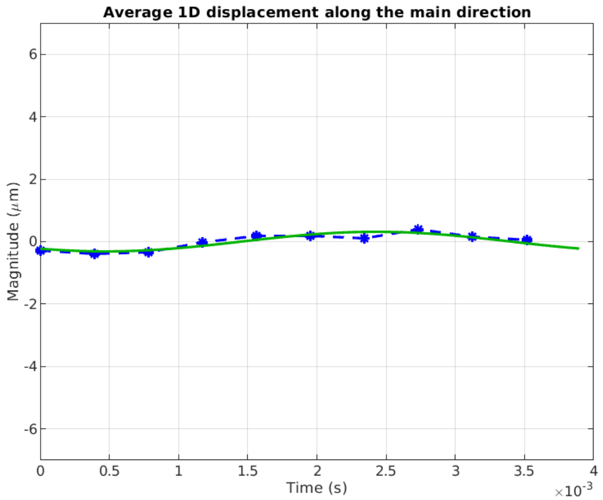

malleus

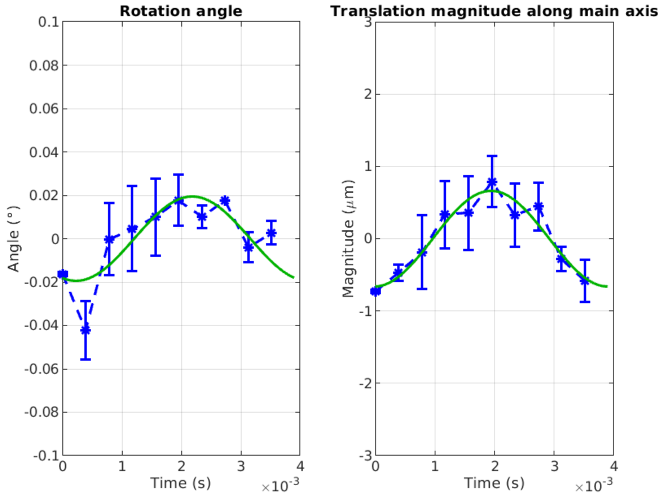

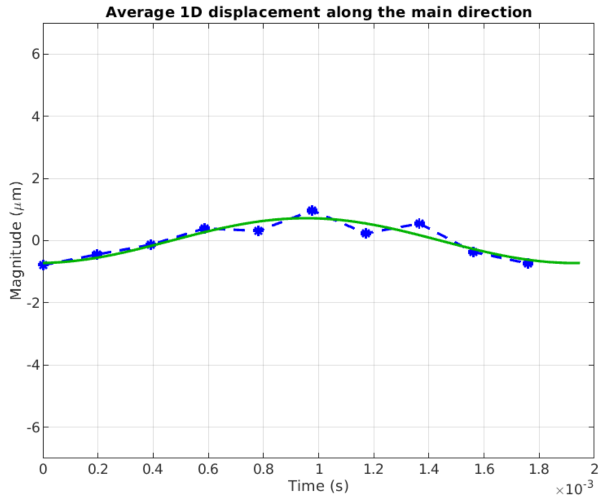

TB1

512 Hz – 110 dB SPL

stapes

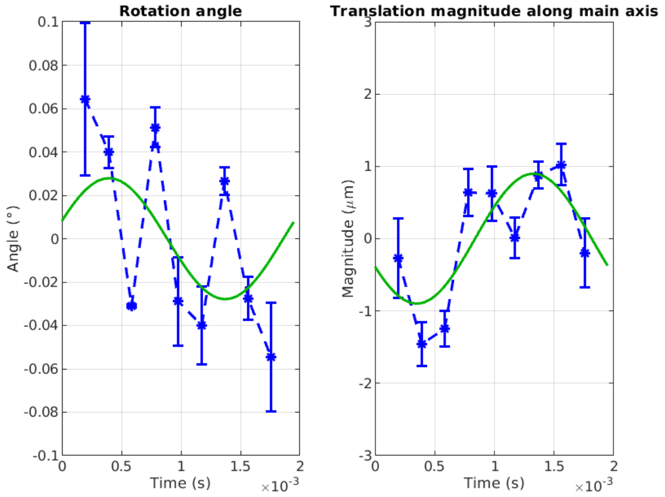

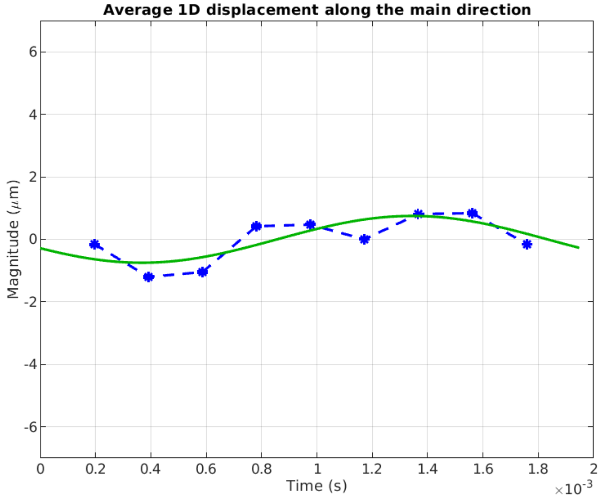

malleus

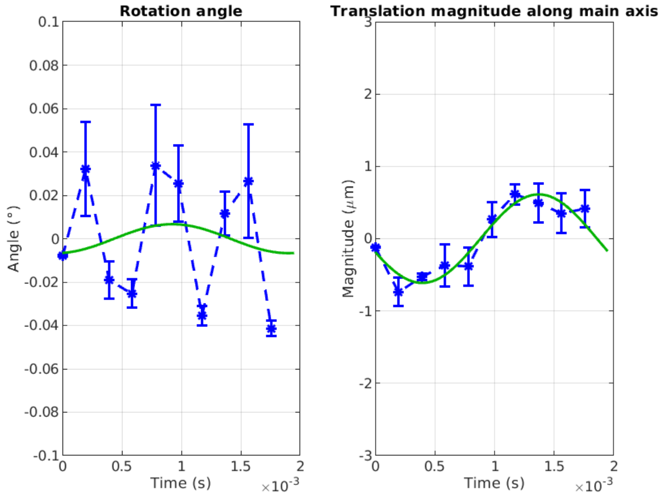

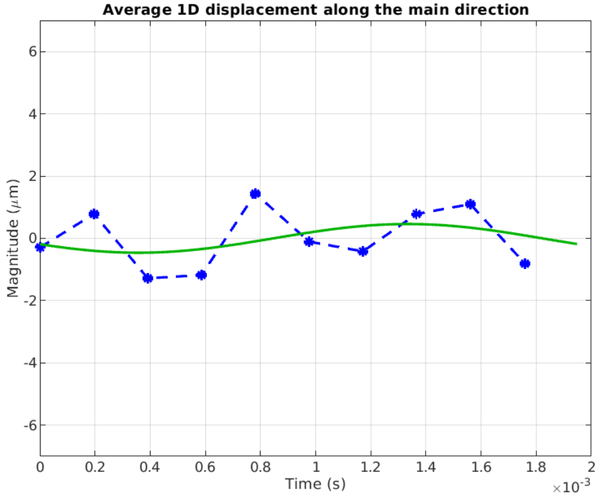

TB1

512 Hz – 120 dB SPL

stapes

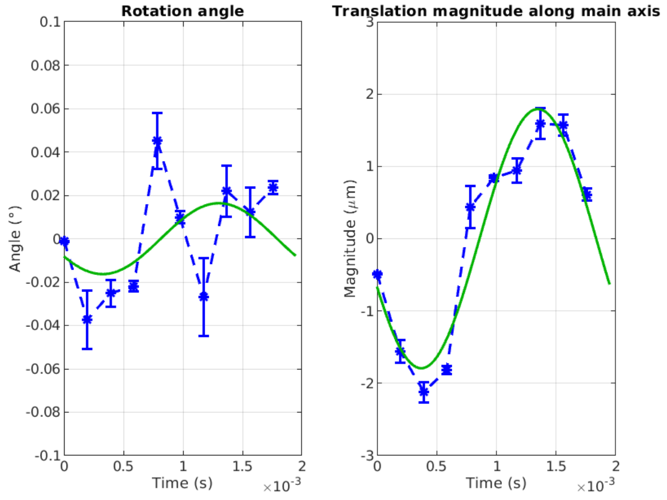

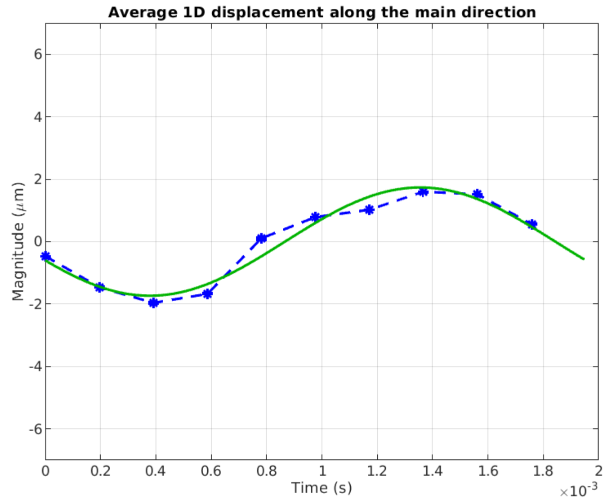

malleus

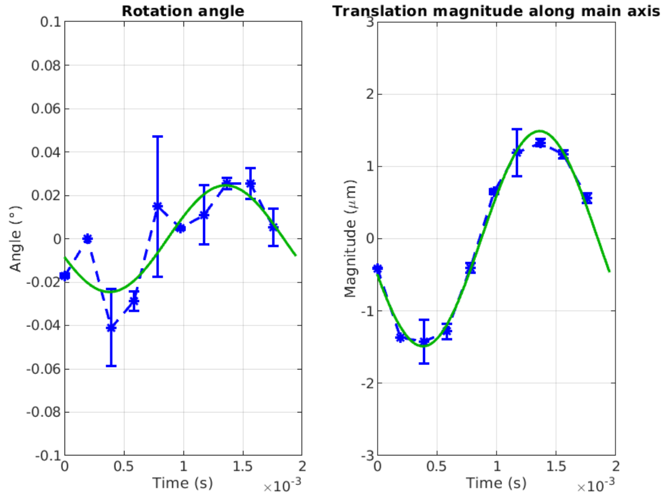

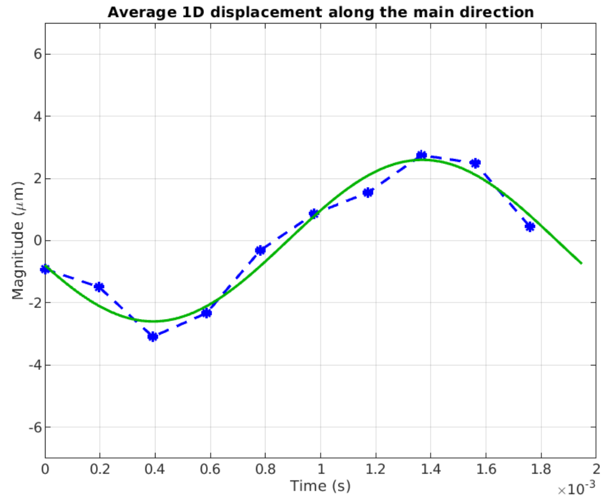

TB2

256 Hz – 110 dB SPL

stapes

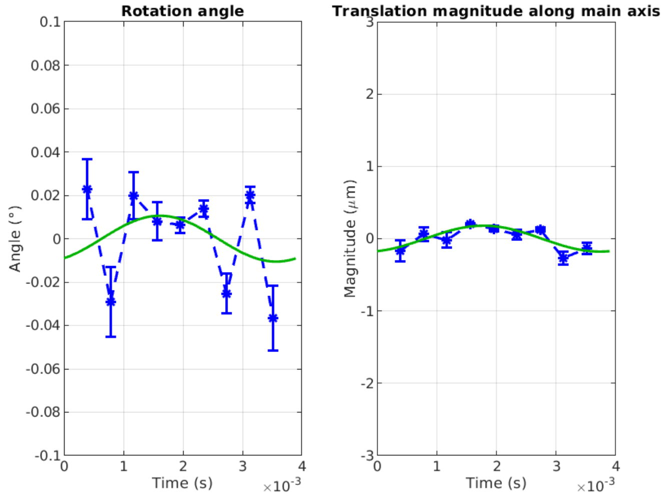

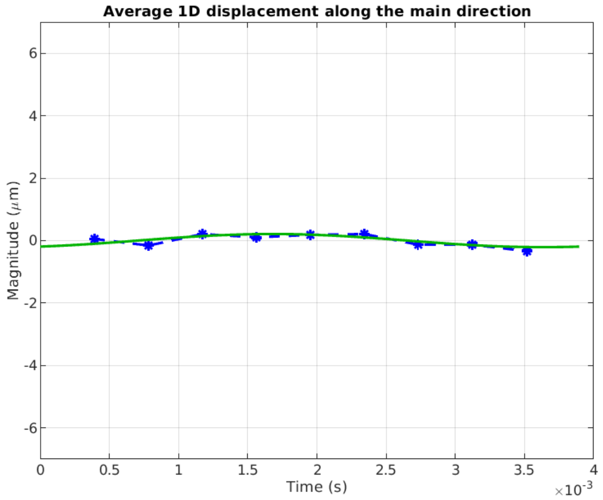

malleus

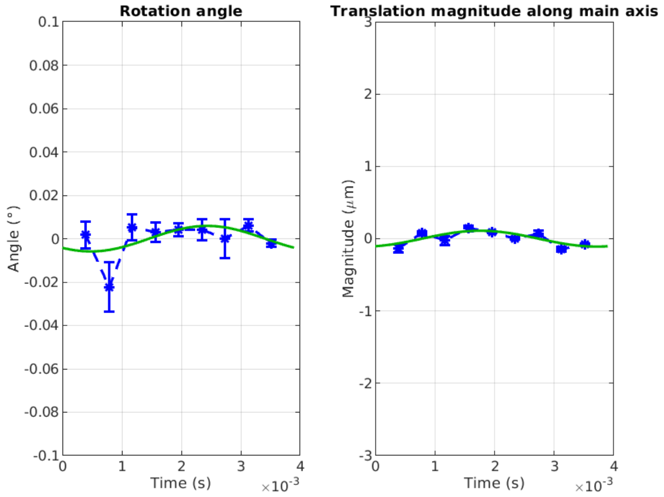

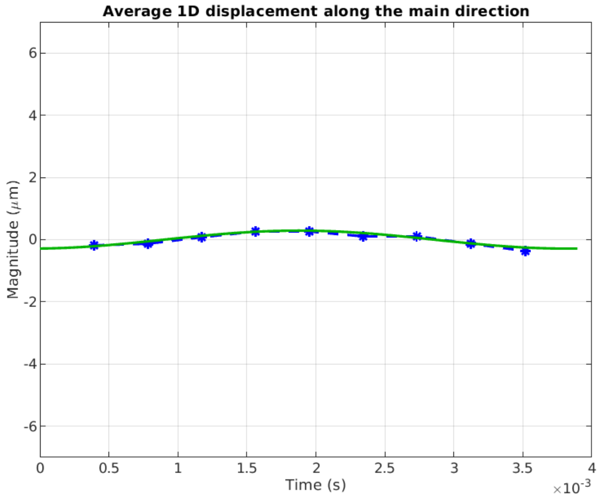

TB2

256 Hz – 120 dB SPL

stapes

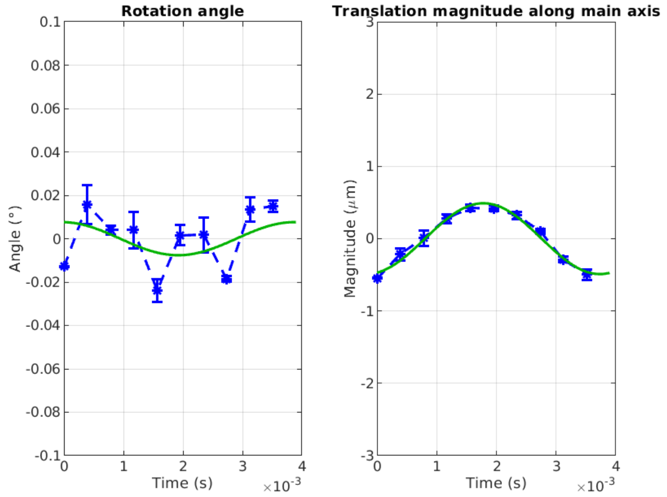

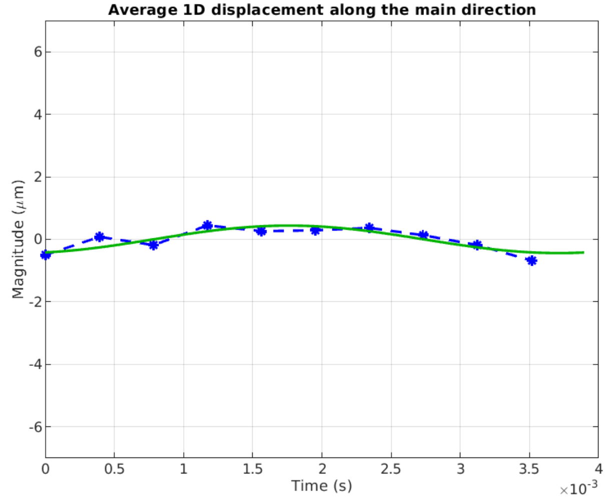

malleus

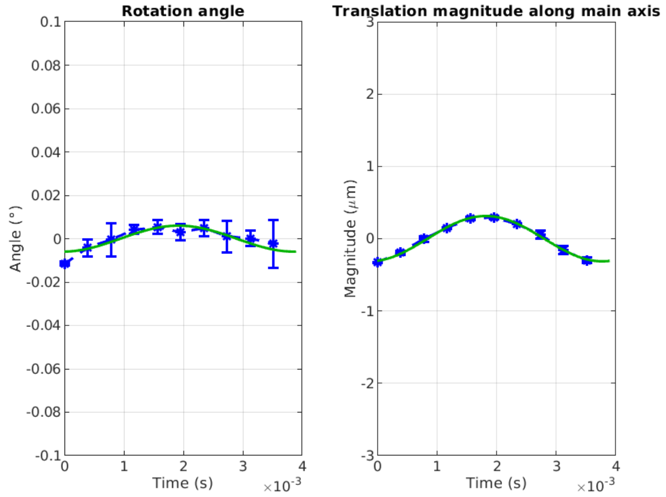

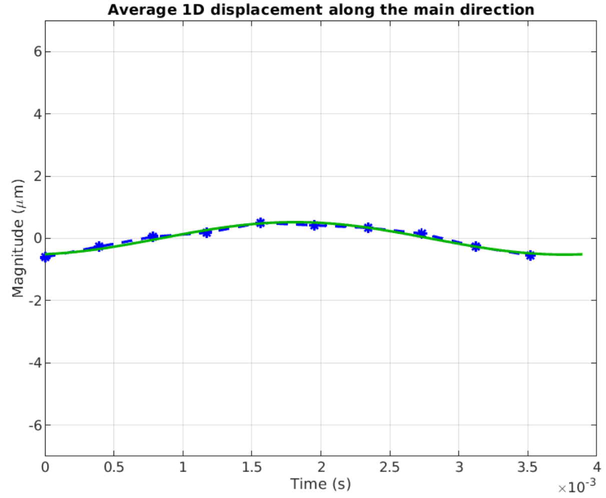

TB2

512 Hz – 110 dB SPL

stapes

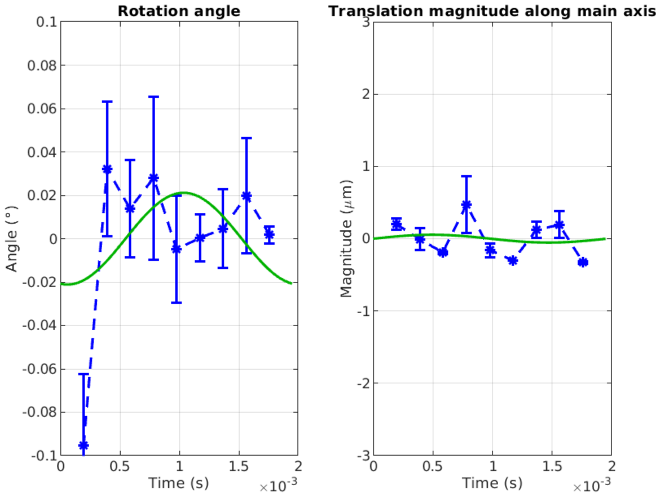

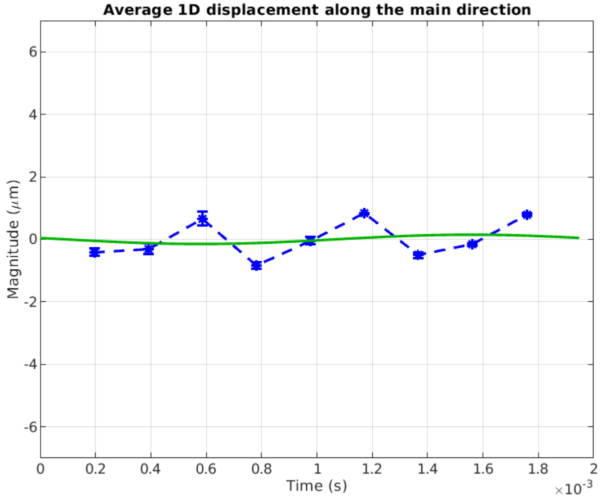

malleus

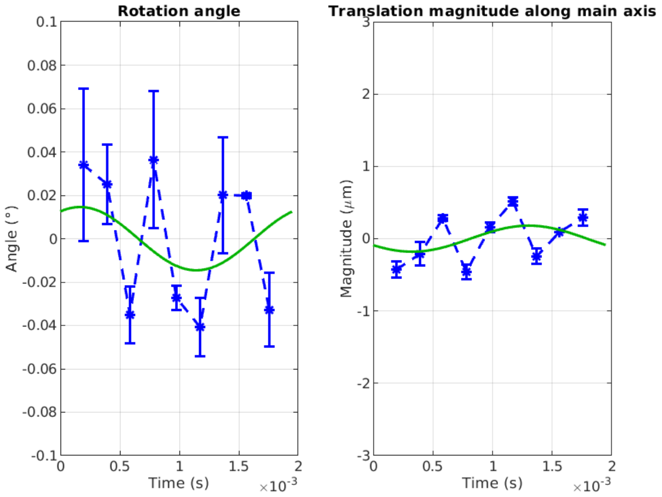

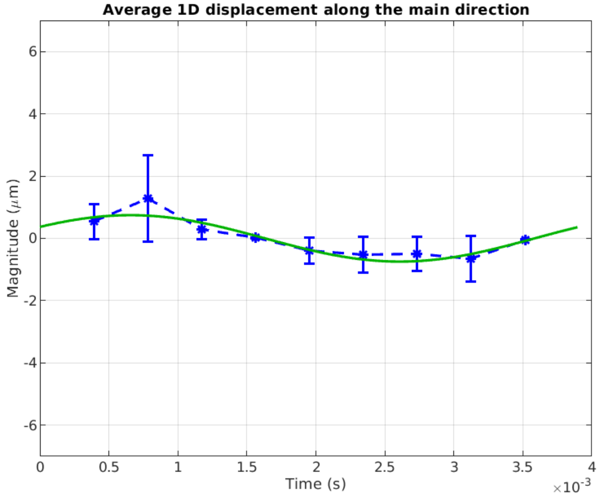

TB2

512 Hz – 120 dB SPL

stapes

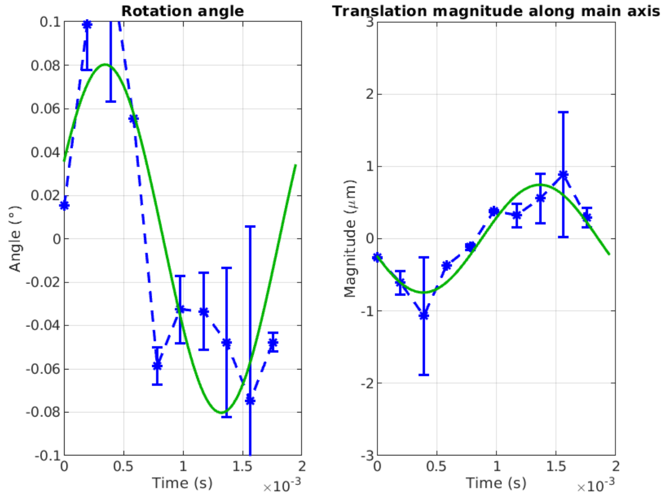

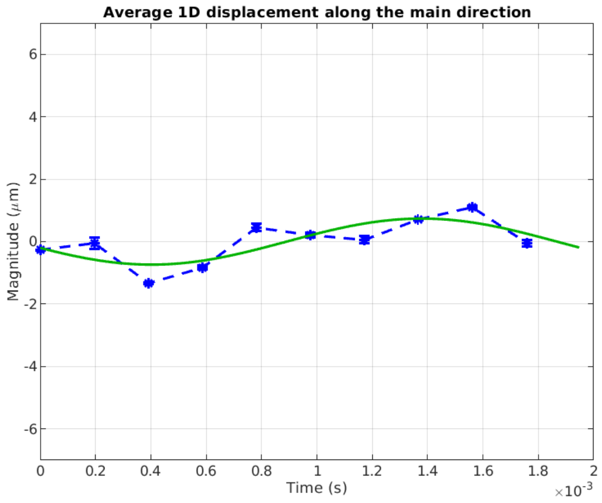

malleus

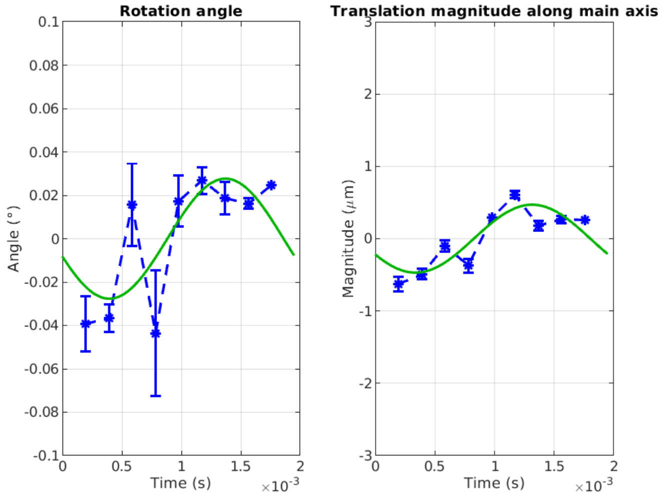

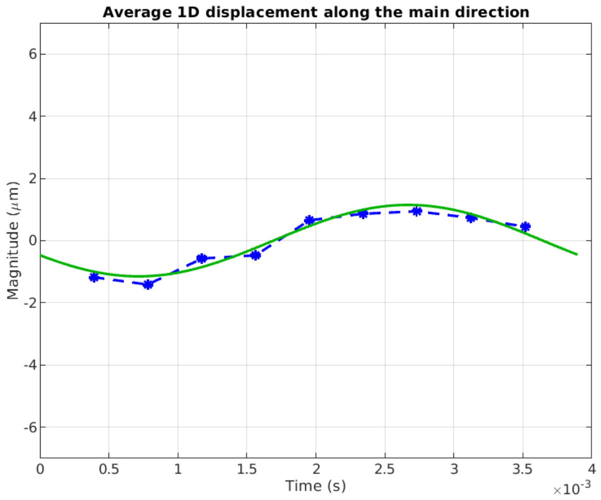

TB3

256 Hz – 110 dB SPL

stapes

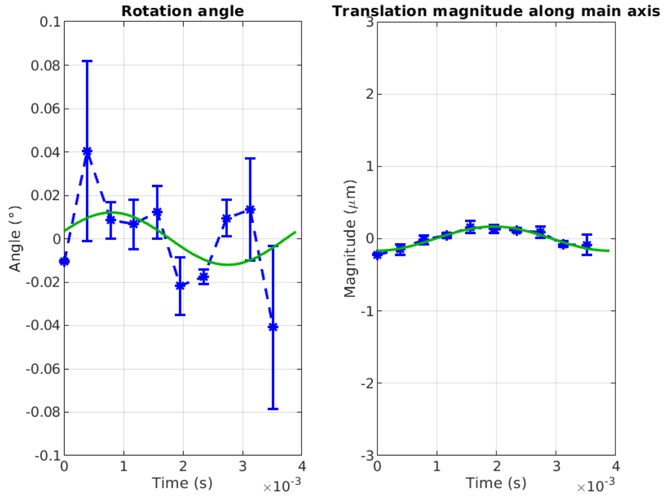

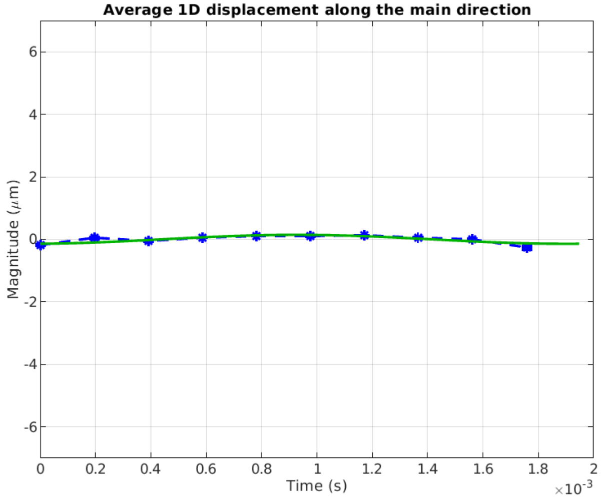

malleus

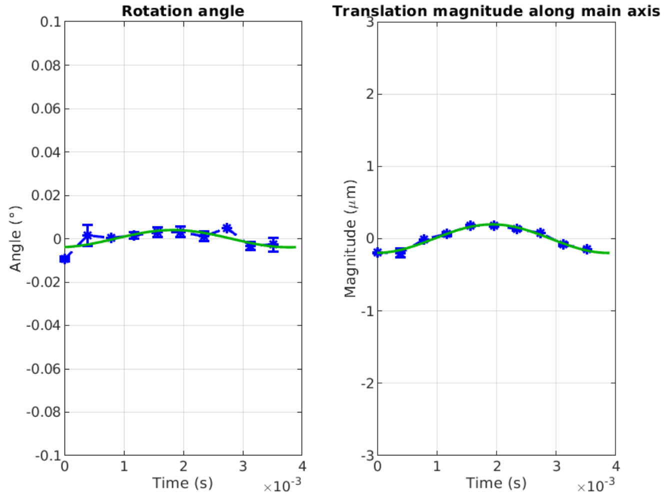

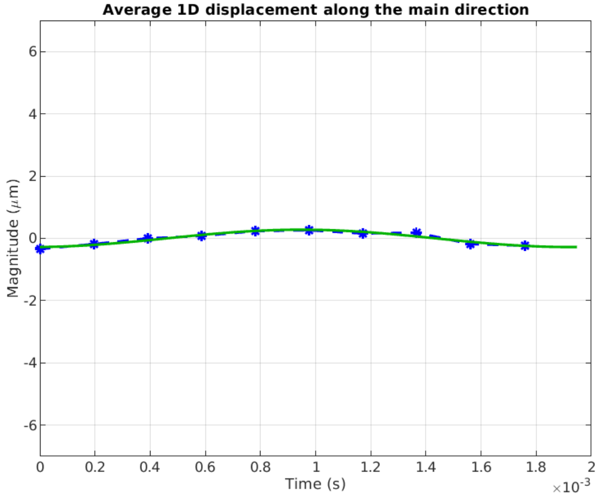

TB3

256 Hz – 120 dB SPL

stapes

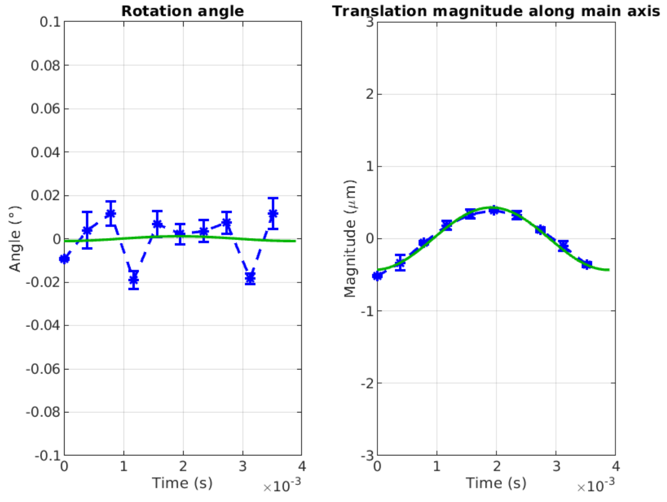

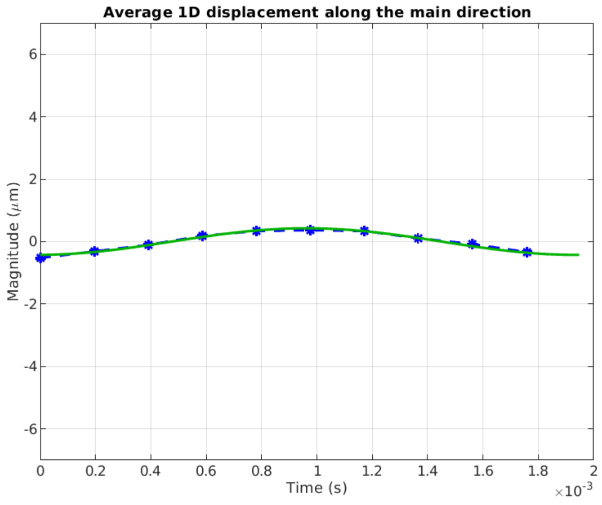

malleus

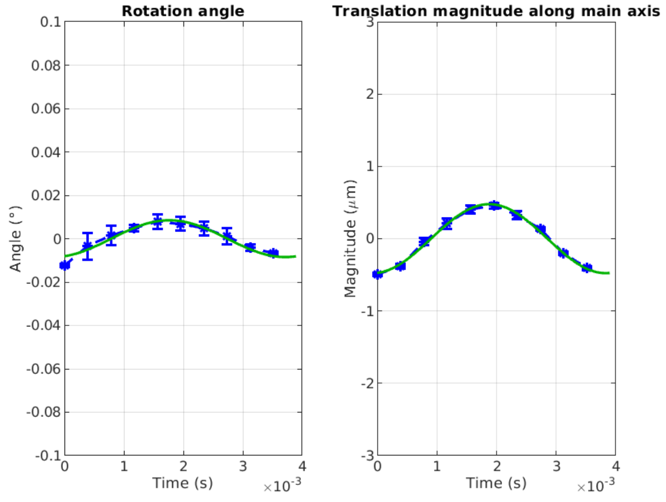

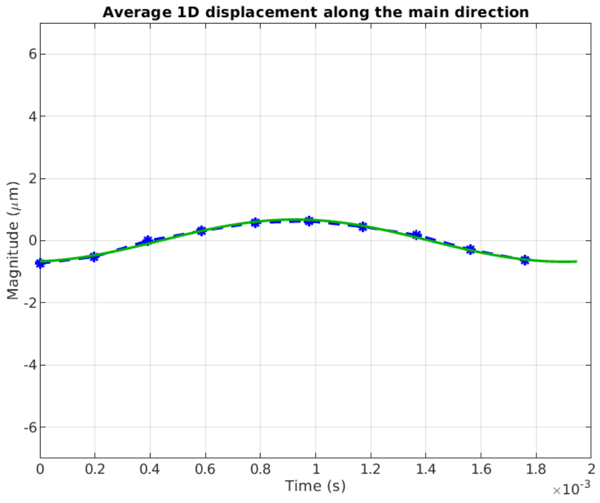

TB3

512 Hz – 110 dB SPL

stapes

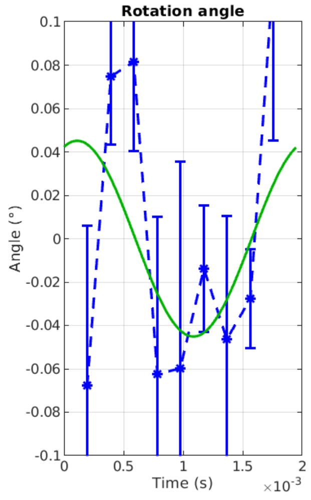

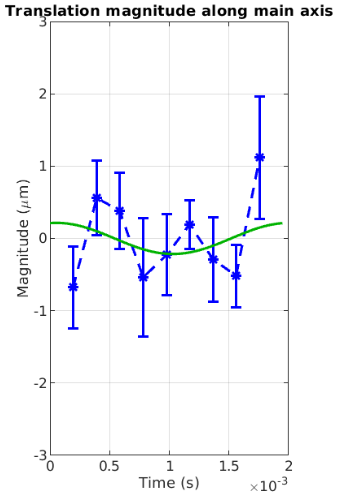

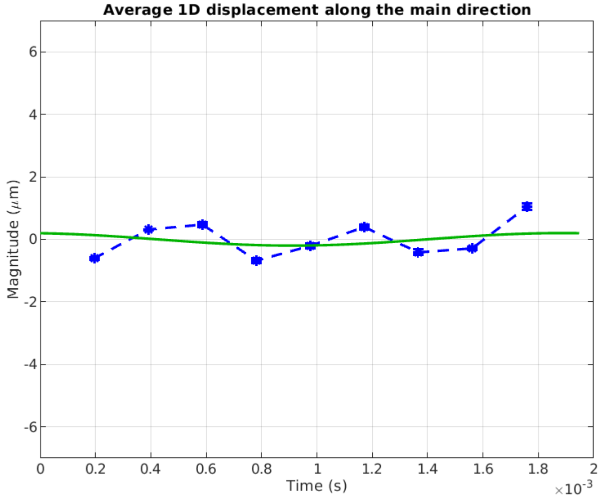

malleus

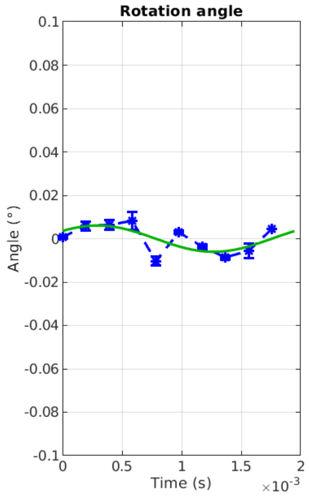

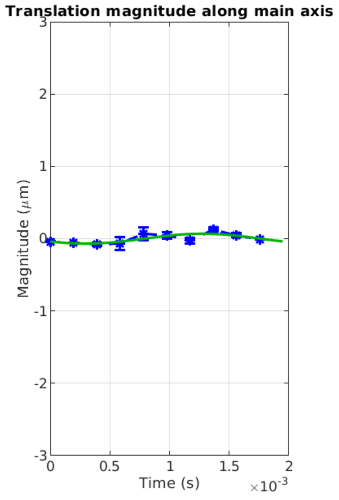

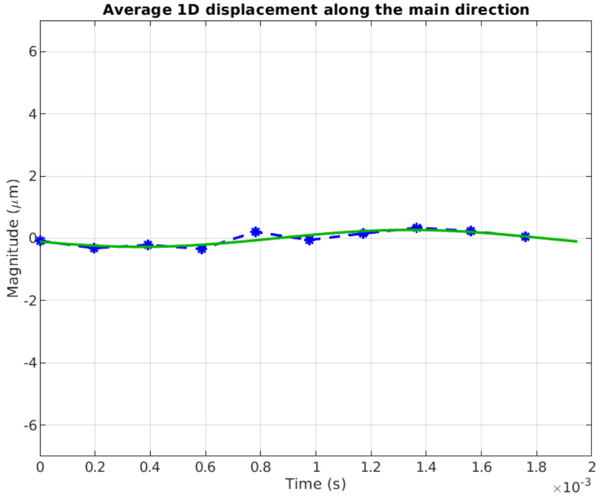

TB3

512 Hz – 120 dB SPL

stapes

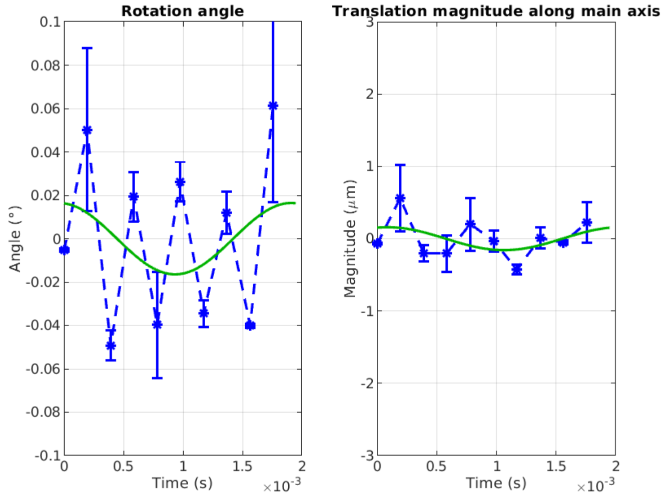

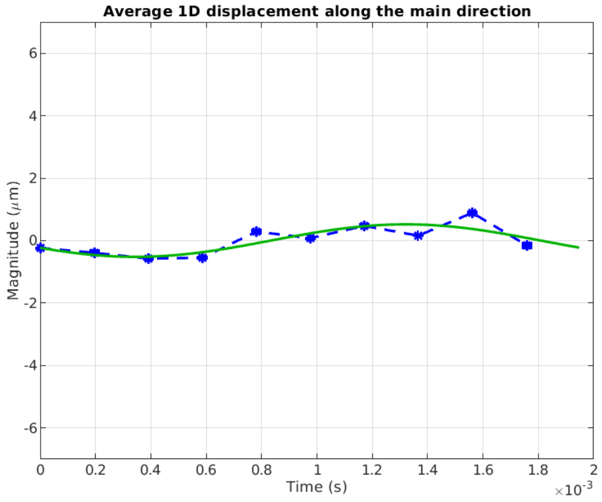

malleus

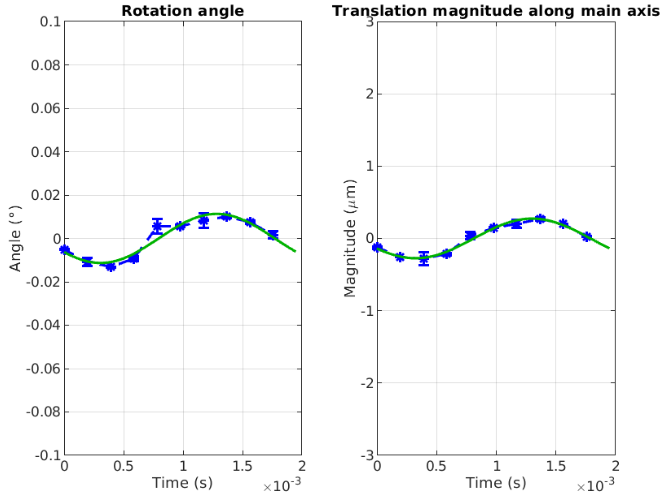

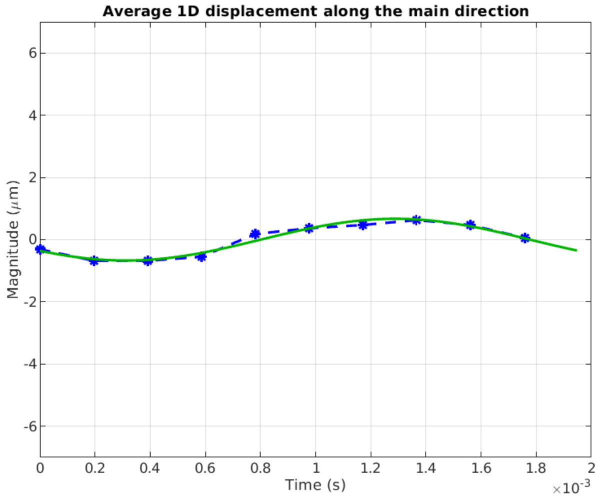

Supplement: Supplementary file 2 — Supplementary file2 (PDF 1414 KB) [file 10162_2024_971_MOESM2_ESM.pdf]
